# Visualization techniques and graphical user interfaces in syndromic surveillance systems. Summary from the Disease Surveillance Workshop, Sept. 11–12, 2007; Bangkok, Thailand

**DOI:** 10.1186/1753-6561-2-s3-s6

**Published:** 2008-11-14

**Authors:** Kieran M Moore, Graham Edge, Andrew R Kurc

**Affiliations:** 1 Department of Emergency Medicine, Family Medicine, Queen's University, 995 Auden Park Drive, Kingston, Ontario, Canada K7M 7T9; 2 Q.U.E.S.S.T c/o Kingston, Frontenac, Lennox & Addington Public Health, 221 Portsmouth Avenue, Kingston, Ontario, Canada, K7M 1V5

## Abstract

Timeliness is a critical asset to the detection of public health threats when using syndromic surveillance systems. In order for epidemiologists to effectively distinguish which events are indicative of a true outbreak, the ability to utilize specific data streams from generalized data summaries is necessary. Taking advantage of graphical user interfaces and visualization capacities of current surveillance systems makes it easier for users to investigate detected anomalies by generating custom graphs, maps, plots, and temporal-spatial analysis of specific syndromes or data sources.

## Background

Although electronic surveillance systems are able to automatically detect statistical anomalies in syndromic data, this creates an alert that needs epidemiological investigation. Human interpretation is required to interpret the alerts and raw data to separate statistically significant but epidemiologically unimportant events from real disease outbreaks. To this end, effective syndromic surveillance systems incorporate a graphical user interface and multiple data visualization techniques in order to aid epidemiologists in deciphering large amounts of data in a timely and cost effective manner. The presence of data tables or line listings allows easy insight into recent system activity. These tables will visually highlight any instances where the data has exceeded the statistically predicted range of values and allow the epidemiologist to confirm any statistical aberration alerts. This allows users to quickly identify potential outbreak situations and also provides them with extra information to further investigate the situation such as the geographic area, population, statistically predicted values, received values, and upper confidence limits. Automatically generated time series plots allow epidemiologists to examine recent trends (monthly, yearly, seasonal) from incoming data streams.

Geospatial visualization of data helps users to identify the significance of any recent data anomalies. By viewing a map generated by an integrated geographic information system, epidemiologists can identify clusters of increased activity or determine if the increases in data are randomly dispersed. Such geospatial visualization can also assist public health in tracking an outbreak, by creating maps of received data overlaid with infrastructure, water sources, or hospital locations.

By making it faster and simpler for epidemiologists to analyze large amounts of data, a well-designed user interface ensures cost effective surveillance and timely outbreak detection.

## Discussion

One of the greatest assets of syndromic surveillance systems is their timeliness. These systems receive data in real-time or near real-time, and thus have the potential to detect changes in the public's health more rapidly than traditional reporting methods. Although these systems can automatically gather, parse, and analyze syndromic data sources, for the most part the alerts generated are merely suggestive, and not conclusive [1]. Human interpretation is needed to examine the data gathered by surveillance systems and to determine when a public health response is warranted. Since these systems can collect data automatically and in real-time, the timeliness of outbreak detection is largely dependant on how long it takes epidemiologists to survey the gathered data and separate real disease outbreaks from false alarms. Furthermore, real disease outbreaks are scarce, and so for syndromic surveillance systems to be cost effective, it is important to reduce as much as possible the time needed for day-to-day monitoring by epidemiologists. To this end, effective surveillance systems incorporate graphical user interfaces and multiple data visualization techniques to make it easy for epidemiologists to examine large quantities of data and routing alerts in a timely and cost effective manner.

**Figure 1:**
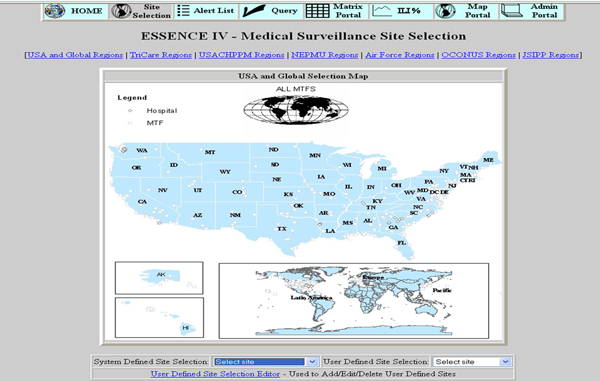
**ESSENCE Medical Surveillance Site Selection Screen**. Figure 1 shows a map of the United States from the ESSENSE Medical Surveillance Site Selection screen where users can gain access to more detailed data on a site of interest.

Syndromic surveillance systems are typically available to epidemiologists over a secure Internet connection. Once logged in to the system, a main page provides a summary of recent system activity and any statistical alerts that have been generated. The main screen for the CDC's BioSense system for example, displays the past week of data for each syndrome monitored, allowing the user to easily identify any syndromes which have recently experienced elevated levels [2]. Also on the main page is a list of data sources and what percentage of expected records were received, providing some indication if any data duplication, increase in reporting sites, or incomplete data delivery has occurred. Selecting a specific syndrome displays additional information, indicating which data sources the excess (potential outbreak) occurred in. The ESSENCE system used by the United States military monitors data from 413 military treatment facilities worldwide, and so users must be able to easily view all regions when accessing the system (Figure 1).

**Figure 2:**
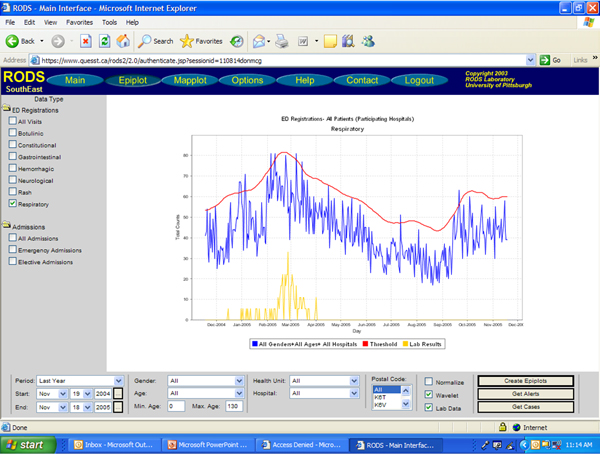
**RODS Epiplot Function of Sample Data – threshold, syndromic counts, and laboratory confirmed cases**. Figure 2 shows an Epiplot of sample influenza data. The red line indicates the threshold level at which an alert would be generated; the blue line indicates respiratory syndromic counts from emergency departments of participating hospitals; the yellow line shows laboratory confirmed cases of influenza.

**Figure 3:**
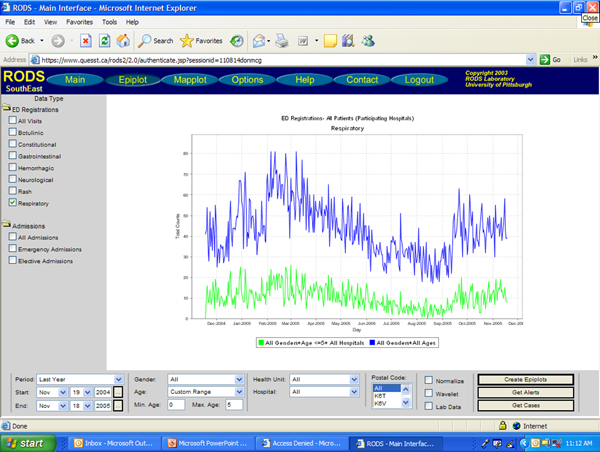
**RODS Epiplot Function of Sample Data – Total Syndromic Counts vs. Age-restricted Subgroup**. Figure 3 shows another example of an Epiplot of the same sample influenza data. The blue line shows respiratory syndromic counts from emergency departments of participating hospitals; the green line indicates those counts from a subset of age-restricted data (in this case <=5 years of age).

In order for users to effectively distinguish which statistical events are indicative of true outbreaks, the ability to "drill down" from generalized data summaries into more specific data streams is necessary. Surveillance systems make it easier for users to investigate the statistically detected anomalies by generating custom time series graphs of specific syndromes or data sources. In determining whether or not a statistical event merits a public health response, these graphs allow the user to view the recent activity of any particular data source and visually interpret the magnitude of the increase which triggered the alarm. For example the RODS system provides this functionality from the Epiplot window, easily accessible from the Main window. The Epiplot window (Figures 2, 3) allows users to select a region, syndrome, and time interval to view, and also allows users to view case-level detail for the displayed time series and to download the selected data as a comma separated file for further analysis [3]. Similarly, ESSENCE provides a "query portal" from which users can select specific data elements to view over a specified time frame, and export selected data elements to Microsoft Excel for offline data analysis [4].

**Figure 4:**
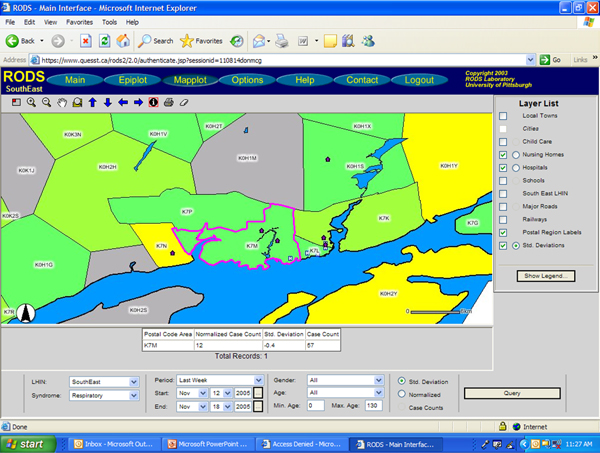
**RODS Mapplot function of sample data – FSA counts colour coded; layers added**. Figure 4 is a Mapplot of the Kingston, Ontario, region by FSA, with a single region selected (outlined in pink). Respiratory syndrome count magnitude, where applicable, is colour coded into FSA boundaries. Additional layers include: nursing home, and hospital locations.

Data submitted to surveillance systems typically includes a geographic identifier such as a ZIP code or in Canada, by postal code. To enhance the interpretation of syndromic data by allowing as special visualization, surveillance systems commonly generate maps with colour gradients denoting the magnitude of received data. If a single data source draws from many smaller regions, it is important to be able to visualize the counts from each region in addition to the whole dataset, so that early cases of an outbreak in a single region are not lost to noise [5]. Many surveillance systems incorporate GIS tools which allow the various data sources to be plotted topographically by a standardized geographical locator and overlaid with other data of interest such as large metropolitan areas, water sources, hospital locations, and highway systems. The mapping layers should include any important infrastructure based on a threat and risk assessment. ESSENCE uses a web-based map view with multiple layers such as emergency room (ER) data, over the counter (OTC) drug sales, and school absenteeism data. These layers can be broken down into sub-layers such as ER data for individual syndromes or absenteeism records from high schools [4]. Accessible from the main window, the RODS Mapplot window (Figure 4) provides an interface to ArcIMS, an internet GIS server developed by ESRI [3]. ArcIMS colours ZIP/postal code regions to show proportions of particular syndromes in the data, and can overlay state/provincial boundaries, water bodies, hospital locations, landmarks, streets, and highways on the public health data [6]. The BioSense main screen provides a map view of any syndrome data selected, or a separate map page can be accessed from the side bar which allows data sources to be transformed before mapping [7].

Certain recently developed surveillance systems have combined temporal and spatial analysis to further facilitate the investigation of data trends and anomalies. The ability to simultaneously view temporal changes and spatial distributions of syndromic data can be helpful in visualizing the propagation of statistically significant events. The SendSS syndrome surveillance module – constructed by the Georgia Division of Public Health – integrates a spatio-temporal visualization interface, which plots semi-transparent circles on a regional map with the circle radius representative of the magnitude of the data [8]. The data presented on the map can be moved forward or backward in time one day at a time, or can be animated to show all daily changes over an interval. The BioPortal Project prototype system includes a Spatial Temporal Visualizer for data visualization [9]. This tool simultaneously presents a periodic spiral graph of user selected granularity (year, month, week, and day), a two-dimensional timeline of data received, and a GIS view of the spatial distribution of the data contained in the selected interval [10]. Presenting all of this data in the same window can make it easier for users to examine seasonal or weekly trends in the data, view temporal trends, and spatially represent a selected interval of data.

The graphical user interfaces (GUI) and visualization techniques can be evaluated by the Framework recommended from the CDC working group for Evaluating Public health Surveillance Systems [11]. The user interface should be acceptable to the epidemiologists and this will be directly related to its simplicity of use, functionality and ongoing educational programs for the end user. Roll based access to a hierarchy of epidemiological tools may enhance acceptance. Automated report generation, data export and drill down capability will also enhance functionality.

Flexibility of the GUI will allow for a static interface for some users, while advanced users can access increased functionality and a more dynamic interface. Advanced users may want adaptable modifiable syndrome classifications, multiple anomaly capabilities for each data set, or integration of multiple data streams. The dependability of the system is integral, and hence the GUI must be able to inform and allow investigation by the end user regarding data flow interruption, quality and missing data. Sustainability of systems is enhanced if the GUI is part of the normal data access and reporting systems of public health authorities, allowing for integration with reportable disease and laboratory based systems. Hence making the syndromic surveillance GUI a module of an integrated public health information system could facilitate acceptance. To ensure representativeness of the system the GUI should be able to allow visualization of complementary data sets such as telehealth, laboratory, emergency department and pharmacy data.

## Conclusion

Recent advances in the complexity of syndromic surveillance systems allow epidemiologists and other users to receive data in real-time, and provide useful visual interfaces to assist in interpretation. Both spatial and temporal data are often available, and the ability to simultaneously view temporal changes and spatial distributions of syndromic data can be helpful to examine seasonal or weekly trends, view temporal trends, and spatially represent a selected interval of data.

## List of abbreviations used

ArcIMS: Arc Internet Map Server; CDC: US Centers for Disease Control and Prevention; ESSENCE: Electronic Surveillance System for the Early Notification of Community-based Epidemics; GIS: Geographic Information System; NCEIDI: National Center of Excellence for Infectious Disease Informatics; RODS: Real-time Outbreak and Disease Surveillance; SendSS: State Electronic Notifiable Disease Surveillance System.

## Author contributions

GE did the literature search and initial draft. AK reviewed the figures and refined the literature search. KM reviewed, edited and modified all drafts and is responsible for final content. KM conceived of the topic and analysis. All authors read and approved the final manuscript.    

## Competing interests

The authors declare that they have no competing interests.
